# Psychiatric symptoms in neurofibromatosis type 2

**DOI:** 10.1192/j.eurpsy.2021.637

**Published:** 2021-08-13

**Authors:** S. Khouadja, R. Melki, S. Younes, L. Zarrouk

**Affiliations:** Psychiatry, University Hospital Of Mahdia, Mahdia, Tunisia

**Keywords:** psychiatry, Neurofibromatosis, psychosis, acoustic neuroma

## Abstract

**Introduction:**

Neurofibromatosis type 2 (NF2) is a rare disorder associated with significant morbidity such as hearing loss that can lead to many psychiatric disorders.

**Objectives:**

Describe the psychiatric symptoms associated to NF2.

**Methods:**

We report the case of a patient admitted to the locked unit of the psychiatric ward for agitation and persecutory delusion and diagnosed with NF2. The data was collected from the patient’s medical file. A review of the literature was performed by selecting articles from PubMed using ‘Psychosis acoustic neuromas’ and ‘Psychosis neurofibromatosis 2’ as key words.

**Results:**

This is the case of a 21-year-old patient who was admitted for behavioral disorders. Our patient had a medical history of a one-sided deafness treated with a hearing prosthesis. He was also followed irregularly by a free-lance psychiatrist. The start of trouble dated back to 3 years marked by behavioral disorders such as fugue, agitation, irritability and sleep disorder. The symptoms worsen in the last 3 months with appearance of hostility and delusion of persecution towards his mother. The patient declines to eat the food that his mother cooked for him and threatened her with a knife. The clinical overview includes delirium, clastic agitation strikes, emotional lability, cerebral ataxia and conjunctival hyperemia. Brain scanner showed an association of bilateral acoustic neuromas, cavernous and intraventricular meningioma. These clinical and radiological signs met the diagnosis for NF2 according to the consensus conference of the National Institute of Health in Bethesda (USA 1988).

**Conclusions:**

The psychiatric symptoms reported in acoustic neuroma patients are usually described as transient.

